# Supplementary material for: Adaptive responses of animals to climate change are most likely insufficient
Source: Nat Commun. 2019 Jul 23;10:3109. doi: 10.1038/s41467-019-10924-4 (PMC6650445; doi:10.1038/s41467-019-10924-4)
Supplement: Supplementary file 1 — Supplementary Information [file 41467_2019_10924_MOESM1_ESM.docx]

**Supplementary Information**

**Radchuk et al.** **Adaptive responses of animals to climate change are most likely insufficient**

**Supplementary Information Guide**

Supplementary Figures (1-17) 2

Supplementary Tables (1-4) 23

Supplementary Note 1 27

Effects of precipitation 27

Heterogeneity among studies 27

Test of publication bias 28

Supplementary References 29

**Supplementary Figures**

Supplementary Figure 1. Data availability for 1413 species in the PRC dataset (‘Phenotypic Response to Climate’ data). For each species, a single horizontal black line indicates availability of data on different climatic factors, trait categories (phenological and morphological) and selection, as well as on the duration of the study (years: <10, 10-20, 20-30 and >30) and the study location: Northern Hemisphere Europe (NHE), Northern Hemisphere America (NHAm), Northern Hemisphere Asia (NHAs) or Southern Hemisphere (SH). Pictograms show the taxon to which a species belongs (Aves, Mammalia, Amphibia, Reptilia, Insecta and Arachnida) and the number next to the pictogram specifies the number of studies for that taxon. The silhouettes were taken from <http://phylopic.org> (license CC BY-SA 3.0). No changes were made to the silhouettes. Illustration credits: Aves - Ferran Sayol, Mammalia - Steven Traver, Amphibia - Emily Willoughby, Reptilia - Mattia Menchetti / Yan Wong, Insecta - M. A. Broussard, and Arachnida – Lafage.

Supplementary Figure 2. Locations of studies in the PRC dataset (‘Phenotypic Response to Climate’, including both studies with and without data on selection) are shown with empty circles, and locations of studies in the PRCS dataset (‘Phenotypic Responses to Climate with Selection data’, a subset with only studies for which selection data were available) are filled with orange colour. The size of the circles is proportional to the duration of the study (in years). Insets show for each dataset the relative coverage of a) different taxa; b) different climatic factors; and c) different trait categories. This map was produced by V. Radchuk using the function map_data() from ggplot2 package in R^1^.

Supplementary Figure 3. Phenotypic responses of species to the amount of precipitation: a) effect of year on precipitation, b) effect of precipitation on trait values and c) weighted mean selection over time (WMSD) acting on traits over the study period. Each study case is identified by a) the country and publication identity (publication identities are given in Supplementary Data 3), b) the trait and publication identity, and c) the trait, fitness measure and publication identity. Only phenological traits were covered by the studies extracted from the publications that focused on the effects of precipitation. The mean effects across studies in the PRCS dataset (‘Phenotypic Responses to Climate with Selection data’, shown in black) and the PRC dataset (‘Phenotypic Response to Climate’, shown in blue) suggest: a) the absence of a directional change of precipitation with time, b) the absence of a relationship between precipitation and trait, and c) evidence for negative selection on phenological traits, with significant variation in WMSD measured using different fitness components (recruitment, reproduction and adult survival).

Supplementary Figure 4. Probability density of the duration (years) of the studies in the PRC dataset (‘Phenotypic Response to Climate’, shown in black) and in the PRCS dataset (‘Phenotypic Responses to Climate with Selection data’, shown in orange). The vertical lines show median study duration in each dataset. Probability densities were smoothed using Gaussian kernel density estimation based on the default bandwidth selection in the geom_density() function (ggplot2 package in R^1^).

Supplementary Figure 5. Linear models explaining the warming rates by a) number of years in the time series and b) first year in the time series, and c) Pearson correlation between the number of years in the time series and the first year in the time series. Warming rates are lower for longer time series, as shown by the significant slope in a) and they are higher for series that started the most recently, as shown by the significant slope in b).

Supplementary Figure 6. Effect of temperature (blue), year (red), and abundance (green) on the phenological traits shown on the y-axis. Effect sizes were obtained from the mixed-effects models including the abundance as an explanatory variable (Equation (5) in Methods). Each study is identified by study identity, trait and species. Studies are sorted by species and within it by study identity. For study identities see Supplementary Data 4. Effects of temperature are generally larger compared to the effects of year and abundance, and negative (i.e. advancement of phenology with increasing temperature).

Supplementary Figure 7. Temporal trend in selection acting on phenological (black) and morphological (grey) traits. Each study is identified by publication identity, trait and fitness component. Bars show 95% confidence intervals and the symbol size is proportional to the study sample size. Across studies, we found no significant directional change in selection on either phenological (Slope = 0.0005±0.0019 (year*SD)^-1^, LRT between the model with and without linear change in selection over years: χ^2^ = 0.09, df = 1, p = 0.764) or morphological traits (Slope = 0.0005 ±0.0008 (year*SD)^-1^, LRT: χ^2^ = 0.46, df = 1, p = 0.497).

Supplementary Figure 8. Product of the weighted mean annual selection differential (WMSD) with the sign of the slopes obtained when assessing condition 2 and 3 (the overall sign of the climate-driven phenotypic change over time). The product, computed as WMSD*sign of slopes for condition 1 and condition 2 ($\beta_{\mathrm{Clim}}$ * $\beta_{\mathrm{Trait}}$), is shown separately for phenological (black) and morphological (grey) traits. This product is positive if selection acts in the same direction as the observed climate-driven trait change over time, indicating adaptive trait change. If this product is negative, the trait change is maladaptive. Each study is identified by publication identity, trait, and fitness component. The studies are sorted by trait category (phenological, morphological), and within it by species, fitness category and publication identity. Repeated labels correspond to either different locations reported in the same publication, or to measurements on different sexes. Across studies, changes in phenological traits were mainly adaptive, whereas there was no consistent change in morphological traits.

Supplementary Figure 9. Distribution of the proportion of studies at risk for 100,000 random values of $\omega^{2}$. Since we do not have $\omega^{2}$ values for each study, we draw those values from a published compilation of $\omega^{2}$ values^2^. Here we represent the proportion of studies for which the estimated observed lag is larger than the estimated critical lag (i.e. population growth $\lambda$ < 1) when one value of $\omega^{2}$ is drawn per study. The distribution shows that the probability that none of study species is at risk is virtually zero (red arrow).

Supplementary Figure 10. Flowchart showing the number of studies included at each stage of the systematic literature review, which in the end resulted in the ‘Phenotypic Response to Climate’ (PRC) dataset. The flowchart follows the structure of PRISMA flow diagrams.

Supplementary Figure 11. Relations between year and temperature for each study (titles above panels indicate authors and study species) in the PRCS (‘Phenotypic Responses to Climate with Selection data’) dataset. pv is the p value of the slope as estimated by LRT. Note that the range of the x- and y-axes can differ among studies.

Continuation Supplementary Figure 11.

Supplementary Figure 12. Relationships between phenological traits (z-scaled) and temperature for each study in the PRCS (‘Phenotypic Responses to Climate with Selection data’) dataset (titles above panels indicate authors and study species). pv is the p value of the slope as estimated by LRT. Note that the range of the x- and y-axes can differ among studies.

Continuation Supplementary Figure 12.

Supplementary Figure 13. Relationships between morphological traits (z-scaled) and temperature for each study in the PRCS (‘Phenotypic Responses to Climate with Selection data’) dataset (titles above panels indicate authors and study species). pv is the p value of the slope as estimated by LRT. Note that the range of the x- and y-axes can differ among studies.

Supplementary Figure 14. Yearly selection differentials (and their CI, shown with bars) measured on phenological traits shown for each study (titles above panels indicate authors and study species). The red solid line shows the estimate of WMSD (weighted mean selection differential) and the shaded polygon indicates ± SE of WMSD. The overlap of the red shaded polygon with 0 indicates that selection is not significantly different from 0. Note that the range of x- and y-axes can differ among studies.

Continuation Supplementary Figure 14.

.


Supplementary Figure 15. Yearly selection differentials (and their CI, shown with bars) measured on morphological traits shown for each study (titles above panels indicate authors and study species). The red solid line shows the estimate of WMSD (weighted mean selection differential) and the shaded polygon indicates 95% CI of the estimate of WMSD. The overlap of the red shaded polygon with 0 indicates that selection is not significantly different from 0. Note that the range of x- and y-axes can differ among studies.

Continuation Supplementary Figure 15.

Supplementary Figure 16. Funnel plots of the effect sizes obtained when testing each of the three conditions with the PRCS dataset. Shown are sample sizes (number of years) and effect sizes: a) an effect of year on temperature, b) an effect of temperature on phenological traits; c) an effect of temperature on morphological traits; d) selection on phenological traits; e) selection on morphological traits. The solid line is a null effect and the dashed line depicts the mean effect size across all the studies for each model. z and p values above each panel show the results of Egger’s test assessing the asymmetry of the funnel plot (p < 0.05 indicates a significant asymmetry signaling a potential publication bias).

Supplementary Figure 17. Funnel plots of the effect sizes obtained when testing two conditions with the PRC dataset. Shown are sample sizes (number of years) and effect sizes: a) an effect of year on temperature, b) an effect of temperature on phenological traits; and c) an effect of temperature on morphological traits. The solid line is a null effect and the dashed line depicts the mean effect size across all the studies for each model. z and p values above each panel show the results of Egger’s test assessing the asymmetry of the funnel plot (p < 0.05 indicates a significant asymmetry signaling a potential publication bias).

**Supplementary tables**

Supplementary Table 1. Heterogeneity estimates for the mixed-effect meta-analyses used in this study. *Q* is the total amount of heterogeneity and the column ‘p value’ indicates whether this amount of heterogeneity is significant, *I*^2^ reflects the proportion of the total heterogeneity due to between-study variance and ranges from 0 to 1, and *H*^2^ is the ratio showing the proportion of observed heterogeneity in relation to what would be expected under the null hypothesis. The heterogeneity estimates are shown for the models fitted to both datasets and for the models testing how sensitive the results are to excluding two studies.

| Data | Response | *I*^2^ | *H*^2^ | *Q* | p value |
| --- | --- | --- | --- | --- | --- |
| PRCS | Slope of temperature on years | 0.640 | 2.8 | 104.8 | <0.0001 |
|  | Slope of phenological traits on temperature | 0.915 | 11.8 | 365.2 | <0.0001 |
|  | Slope of morphological traits on temperature | 0.000 | 1.0 | 6.4 | 0.7024 |
|  | Mean selection across phenological traits (presumably induced by temperature) | 1.000 | 120425.2 | 413110.5 | <0.0001 |
|  | Mean selection across morphological traits (presumably induced by temperature) | 0.940 | 16.8 | 178.1 | <0.0001 |
|  | Slope of precipitation on years | 0.002 | 1.0 | 6.0 | 0.7377 |
|  | Slope of phenological traits on precipitation | 0.750 | 4.0 | 20.5 | 0.0085 |
|  | Mean selection across phenological traits (presumably induced by precipitation) | 0.971 | 34.1 | 348.9 | <0.0001 |
| PRC | Slope of temperature on years | 0.542 | 2.2 | 2038.6 | <0.0001 |
|  | Slope of phenological traits on temperature | 0.716 | 3.5 | 13818.1 | <0.0001 |
|  | Slope of morphological traits on temperature | 0.301 | 1.4 | 193.2 | 0.0001 |
|  | Slope of precipitation on years | 0.211 | 1.3 | 50.2 | 0.3857 |
|  | Slope of phenological traits on precipitation | 0.634 | 2.7 | 762.6 | <0.0001 |
| Sensitivity analysis, PRCS dataset | Slope of phenological traits on temperature after removing an outlier (Goodenough et al. 2011)^3^ | 0.914 | 11.7 | 360.1 | <0.0001 |
|  | Mean selection across phenological traits (presumably induced by temperature) after removing an outlier (Goodenough et al. 2011)^3^ | 1.000 | 38169.4 | 412972.2 | <0.0001 |
|  | Slope of phenological traits on temperature after removing the only study on mammal (Plard et al. 2014)^4^ | 0.916 | 11.9 | 356.3 | <0.0001 |
|  | Mean selection across phenological traits (presumably induced by temperature) after removing the only study on mammal (Plard et al. 2014)^4^ | 1.000 | 48625.2 | 408249.8 | <0.0001 |
|  | Slope of phenological traits on temperature after removing both the study on mammal (Plard et al. 2014)^4^ and an outlier (Goodenough et al. 2011)^3^ | 0.915 | 11.7 | 351.3 | <0.0001 |
|  | Mean selection across phenological traits (presumably induced by temperature) after removing both the study on mammal (Plard et al. 2014)^4^ and an outlier (Goodenough et al. 2011)^3^ | 1.000 | 15415.8 | 408111.7 | <0.0001 |

Supplementary Table 2. Sensitivity analyses assessing how the estimates of mixed-effects meta-analytical models fitted to the PRCS (‘Phenotypic Responses to Climate with Selection data’) dataset were affected when excluding 1) the outlier-like study ^3^, 2) the only study on mammals ^4^, and 3) both of them. Given are the model estimates for each model and their standard errors (SE) with the ‘Response’ column specifying the response variable and the ‘Effect’ column specifying the tested effect. For details on the levels of the fixed effects see Methods.

| Excluding | Response | Effect | Modality | Estimate | SE |
| --- | --- | --- | --- | --- | --- |
| The study by Goodenough et al. (2011) ^3^, outlier | Slope of phenological traits on temperature | Intercept |  | -0.239 | 0.067 |
|  | Mean selection across phenological traits separately (presumably induced by temperature) | Intercept |  | -0.114 | 0.022 |
|  |  | Fitness component | Recruitment | -0.185 | 0.032 |
|  |  |  | Reproduction | -0.091 | 0.028 |
|  |  |  | Survival | -0.020 | 0.042 |
|  |  | Generation length |  | -0.014 | 0.005 |
| The only study on mammal (Plard et al. 2014) ^4^ | Slope of phenological traits on temperature | Intercept |  | -0.276 | 0.070 |
|  | Mean selection across phenological traits separately (presumably induced by temperature) | Intercept |  | -0.162 | 0.065 |
|  |  | Fitness component | Recruitment | -0.235 | 0.076 |
|  |  |  | Reproduction | -0.149 | 0.069 |
|  |  |  | Survival | -0.064 | 0.084 |
|  |  | Generation length |  | -0.019 | 0.011 |
| Both the only study on mammal (Plard et al. 2014) ^4^ and the outlier study (Goodenough et al. 2011)^3^ | Slope of phenological traits on temperature | Intercept |  | -0.256 | 0.068 |
|  | Mean selection across phenological traits separately (presumably induced by temperature) | Intercept |  | -0.113 | 0.023 |
|  |  | Fitness component | Recruitment | -0.181 | 0.031 |
|  |  |  | Reproduction | -0.090 | 0.027 |
|  |  |  | Survival | 0.008 | 0.046 |
|  |  | Generation length |  | -0.014 | 0.005 |

Supplementary Table 3. Sensitivity analyses assessing how the significance of the effects obtained with the mixed-effects meta-analytical models fitted to the PRCS (‘Phenotypic Responses to Climate with Selection data’) dataset was affected when excluding 1) the outlier-like study ^3^, 2) the only study on mammals ^4^, and 3) both of them. The significance was estimated with asymptotic likelihood ratio chi-square test (LRT) comparing the model that includes the effect specified in the column ‘Effect’ to the model without this effect. Significant effects (p < 0.05) are highlighted in bold and marginally significant (p < 0.10) in italic. For each model we also show the variance of the random effects: study and publication identity.

| Excluding | Response | Effect | LRT | df | p value | Random variance due to study ID | Random variance due to publication ID |
| --- | --- | --- | --- | --- | --- | --- | --- |
| The study by Goodenough et al. (2011)^3^, outlier | Slope of phenological traits on temperature | **Intercept** | **10.10** | **1** | **0.0015** | 4.46E-02 | 3.94E-02 |
|  | Mean selection across phenological traits separately (presumably induced by temperature) | **Intercept** | **14.47** | **1** | **0.0001** | 9.68E-03 | 2.08E-03 |
|  |  | **Fitness component** | **10.65** | **2** | **0.0049** | 5.49E-03 | 3.39E-03 |
|  |  | Generation length | 0.39 | 1 | 0.5331 | 9.15E-03 | 7.15E-03 |
| The only study on mammal (Plard et al. 2014)^4^ | Slope of phenological traits on temperature | **Intercept** | **11.93** | **1** | **0.0006** | 4.33E-02 | 4.66E-02 |
|  | Mean selection across phenological traits separately (presumably induced by temperature) | **Intercept** | **5.58** | **1** | **0.0182** | 9.78E-03 | 6.31E-02 |
|  |  | **Fitness component** | **6.42** | **2** | **0.0404** | 6.74E-03 | 6.57E-02 |
|  |  | Generation length | 0.38 | 1 | 0.5384 | 9.69E-03 | 7.69E-02 |
| Both the only study on mammal (Plard et al. 2014)^4^ and the outlier study (Goodenough et al. 2011)^3^ | Slope of phenological traits on temperature | **Intercept** | **10.79** | **1** | **0.0010** | 4.38E-02 | 3.97E-02 |
|  | Mean selection across phenological traits separately (presumably induced by temperature) | **Intercept** | **13.00** | **1** | **0.0003** | 9.92E-03 | 2.34E-03 |
|  |  | **Fitness component** | **12.41** | **2** | **0.0020** | 5.48E-03 | 2.95E-03 |
|  |  | Generation length | 0.34 | 1 | 0.5602 | 9.16E-03 | 7.40E-03 |

Supplementary Table 4. Baseline parameter values (used if not specified otherwise) and parameter ranges used to assess the sensitivity of the difference between actual and critical lags. The sources for the parameter values are also given.

| Parameter | Baseline value | Range | Data source |
| --- | --- | --- | --- |
| Heritability, *h*^2^ | 0.15 | 0.04 – 0.33 | PRCS dataset |
| The width of the fitness function, $\boldsymbol{\omega}^{\mathbf{2}}$ | 10 | 3 – 50 | Estes and Arnold (2007)^2^, based on the dataset from Kingsolver et al. (2001)^5^ |
| Effective population size, $\boldsymbol{N}_{\mathbf{e}}$ | 100 | 1 – 10000 | Frankham (1995)^6^ |
| Maximum number of offspring produced per individual, *B* | 1.6 | 1.2 – 2 | Gienapp et al. (2012)^7^ |
| Stochastic variation of the phenotypic optimum around the (linear) trend, $\boldsymbol{\sigma}_{\boldsymbol{\theta}}^{\mathbf{2}}$ | 0 | 0 | Because of the lack of data for assessing this parameter^7^ we assumed no environmental variability, with the caveat that our analyses are over-optimistic about the fate of populations |
| Linear selection differential, $\boldsymbol{\beta}$ | 0.171 | 0.01 – 0.0351 | Mean±2SE obtained for WMS in this study |

**Supplementary Note 1**

**Effects of precipitation**

No systematic change across years and studies was observed for precipitation in either dataset (Supplementary Fig. 3a). This finding is contrary to the increase in precipitation recorded over the last decades in the Northern Hemisphere^8,9^. Such a discrepancy may be related to high local variation in precipitation^8^ combined with the small number of studies focusing on this climatic variable in our dataset. The dataset focusing on the effects of precipitation consisted of phenological traits only. We did not find a significant association between precipitation and phenological traits in both datasets (Supplementary Fig. 3b, Supplementary Data 2). In the dataset investigating effects of precipitation on traits, we found marginally significant negative weighted mean annual selection differential over time (WMSD = -0.101± 0.048 SD^-1^; LRT between the model assuming WMSD is non-zero and the one assuming it equals zero: χ^2^ = 3.2, df = 1, p = 0.072). We found significant variation in selection among fitness components with most negative selection found for recruitment (Supplementary Data 1).

**Heterogeneity among studies**

The meta-analytical models differed in the amount of heterogeneity among studies (Supplementary Table 1). For models focusing on the effects of temperature, the amount of heterogeneity was moderate (Higgins *I*^2^ between 0.3 and 0.6) in the models describing the change in temperature over years and the change of morphological traits with temperature in the PRC dataset. The amount of heterogeneity was substantial (Higgins *I*^2^ between 0.6 and 0.75) for the model assessing the change of temperature over years in the PRCS dataset and the model of association between temperature and phenological traits in the PRC dataset. We found considerable heterogeneity (Higgins *I*^2^ > 0.75) for models assessing WMSD on both phenological and morphological traits and the model of the relationship between temperature and phenological traits in the PRCS dataset. The amount of heterogeneity was low in the model describing the association of temperature and morphological traits in the PRCS dataset.

For models focussing on the effects of precipitation, the amount of heterogeneity was low in the models testing the change in precipitation over years in both datasets (Supplementary Table 1), but it was substantial (*I*^2^ > 0.6) in the models describing the association of traits with precipitation in both datasets and the WMSD.

**Test of publication bias**

We have not found evidence of the small-study effect for the effect sizes used to assess all three conditions with the PRCS dataset (Supplementary Fig. 16). For the effect sizes obtained with the PRC dataset, no evidence of the small-study effect was found when testing condition 2 (Supplementary Fig. 17b,c). However, we found funnel plot asymmetry when testing condition 1 (Supplementary Fig. 17a, Egger’s test: z = 5.88, p < 0.001). Such evidence of plot asymmetry is unlikely to result from a publication bias in this particular case. Instead, as we show in Supplementary Fig. 5, studies spanning shorter period of time are associated with faster rate of climate change because 1) they correspond to more recent studies, and 2) the pace of climate change has been increasing. Interestingly, the fact that shorter time series revealed higher rates of warming is in line with the recent study^10^ reporting the same findings.

**Supplementary References**

1. Wikham, H. *ggplot2: Elegant Graphics for Data Analysis*. (Springer-Verlag, 2016).

2. Estes, S. & Arnold, S. J. Resolving the paradox of stasis: Models with stabilizing selection explain evolutionary divergence on all timescales. *Am. Nat.* **169**, 227–244 (2007).

3. Goodenough, A. E., Hart, A. G. & Elliot, S. L. What prevents phenological adjustment to climate change in migrant bird species? Evidence against the ‘arrival constraint’ hypothesis. *Int. J. Biometeorol.* **55**, 97–102 (2011).

4. Plard, F. *et al.* Mismatch between birth date and vegetation phenology slows the demography of roe deer. *PLoS Biol.* **12**, e1001828 (2014).

5. Kingsolver, J. G. *et al.* The strength of phenotypic selection in natural populations. *Am. Nat.* **157**, 245–261 (2001).

6. Frankham, R. Effective population size/adult population size ratios in wildlife: A review. *Genetics Research* **89**, 491–503 (2008).

7. Gienapp, P. *et al.* Predicting demographically sustainable rates of adaptation: can great tit breeding time keep pace with climate change? *Philos. Trans. R. Soc. B Biol. Sci.* **368**, 20120289 (2012).

8. Folland, C. K., Karl, T. R. & Salinger, M. J. Observed climate variability and change. *Weather* **57**, 269–278 (2002).

9. IPCC. *Climate Change 2014: Synthesis Report*. *Contribution of Working Groups I, II and III to the Fifth Assessment Report of the Intergovernmental Panel on Climate Change. In: Core Writing Team, Pachauri RK, Meyer LA (eds) IPCC, Geneva, Switzerland, 151 p.* (2014). doi:10.1017/CBO9781107415324

10. Post, E., Steinman, B. A. & Mann, M. E. Acceleration of phenological advance and warming with latitude over the past century. *Sci. Rep.* **8**, 3927 (2018).
