# Supplementary material for: Adaptive responses of animals to climate change are most likely insufficient
Source: Nat Commun. 2019 Jul 23;10:3109. doi: 10.1038/s41467-019-10924-4 (PMC6650445; doi:10.1038/s41467-019-10924-4)
Supplement: Supplementary file 3 — Reporting Summary [file 41467_2019_10924_MOESM3_ESM.pdf]

nature research

Corresponding author(s): Viktoriia Radhcuk

Last updated by author(s): Apr 15, 2019

Reporting Summary

Nature Research wishes to improve the reproducibility of the work that we publish. This form provides structure for consistency and transparency in reporting. For further information on Nature Research policies, seeAuthors & Referees and theEditorial Policy Checklist .

Statistics

For all statistical analyses, confirm that the following items are present in the figure legend, table legend, main text, or Methods section.

n/a	Confirmed	
☐	☒	The exact sample size (*n*) for each experimental group/condition, given as a discrete number and unit of measurement
☒	☐	A statement on whether measurements were taken from distinct samples or whether the same sample was measured repeatedly
☐	☒	The statistical test(s) used AND whether they are one- or two-sided<br>*Only common tests should be described solely by name; describe more complex techniques in the Methods section.*
☐	☒	A description of all covariates tested
☐	☒	A description of any assumptions or corrections, such as tests of normality and adjustment for multiple comparisons
☐	☒	A full description of the statistical parameters including central tendency (e.g. means) or other basic estimates (e.g. regression coefficient) AND variation (e.g. standard deviation) or associated estimates of uncertainty (e.g. confidence intervals)
☐	☒	For null hypothesis testing, the test statistic (e.g. *F*, *t*, *r*) with confidence intervals, effect sizes, degrees of freedom and *P* value noted<br>*Give P values as exact values whenever suitable.*
☒	☐	For Bayesian analysis, information on the choice of priors and Markov chain Monte Carlo settings
☐	☒	For hierarchical and complex designs, identification of the appropriate level for tests and full reporting of outcomes
☐	☒	Estimates of effect sizes (e.g. Cohen's *d*, Pearson's *r*), indicating how they were calculated

Our web collection on statistics for biologists contains articles on many of the points above.

Software and code

Policy information about availability of computer code

Data collection	WebPlotDigitiser, metagear package in R software
Data analysis	R 3.5.0

For manuscripts utilizing custom algorithms or software that are central to the research but not yet described in published literature, software must be made available to editors/reviewers. We strongly encourage code deposition in a community repository (e.g. GitHub). See the Nature Research guidelines for submitting code & software for further information.

Data

Policy information about availability of data

All manuscripts must include a data availability statement. This statement should provide the following information, where applicable:

- Accession codes, unique identifiers, or web links for publicly available datasets
- A list of figures that have associated raw data
- A description of any restrictions on data availability

The PRC dataset, containing metadata on each study as well as coefficients estimated for each step of the framework (Fig. 1) per each study is available as Supplementary Data 4. We also make available the raw data for a subset of studies for which we received the consent of data owners (4819 out of 4835 studies). These data are available as part of the R package 'adRes' (www.github.com/radchukv/adRes), which implements the complete workflow of this study and provides functions to be used to conduct similar analyses on new data in the future. The source data underlying Figs 1b-c, 1e-f, 2, 3, 4, and 5a-c are provided as a Source Data file.

Field-specific reporting

Please select the one below that is the best fit for your research. If you are not sure, read the appropriate sections before making your selection.

☐ Life sciences ☐ Behavioural & social sciences ☒ Ecological, evolutionary & environmental sciences

For a reference copy of the document with all sections, see nature.com/documents/nr-reporting-summary-flat.pdf

Ecological, evolutionary & environmental sciences study design

All studies must disclose on these points even when the disclosure is negative.

Study description	This study is a meta-analysis based on a systematic literature review. We addressed a question of to what extent the trait changes in animals in response to climate change are adaptive.
Research sample	We focused on adaptive responses of animals, and therefore our search targeted a wide range of animal taxa, from insects to mammals. As a result of our literature search we assembled a dataset consisting of 4835 studies (representing 1413 non-aquatic species in 23 countries). This 'Phenotypic responses to Climate' (PRC) dataset contained information on phenotypic responses to climate change. Out of these 4835 studies a subset of 71 studies (representing 17 species in 13 countries) contained all the information required to assess whether responses were adaptive and was used for the analyses for which selection differentials were necessary.
Sampling strategy	Our sample size was a result of what was available on this topic in the published literature.
Data collection	The data were collected by conducting a systematic literature review. We identified the potentially suitable papers by key words search on Web of Knowledge. In the next step, the 10090 identified abstracts were skimmed and the papers that seemed to satisfy all the necessary requirements were retained. Finally, after the full read of the subselected 180 papers, 58 of them were retained to assemble the dataset.
Timing and spatial scale	The key word search was conducted on May 23rd 2016 in Berlin and the abstract skimming took place in the following months.
Data exclusions	No data were excluded from the analysis
Reproducibility	Not applicable because this is not an experimental study
Randomization	Not applicable. We did not experiment with animals. Instead, our dataset was based on the studies that were published in the literature
Blinding	Blinding is not relevant to our study because we do not experiment with animals but collected the data from studies conducted previously and published in literature.

Did the study involve field work? ☐ Yes ☒ No

Reporting for specific materials, systems and methods

We require information from authors about some types of materials, experimental systems and methods used in many studies. Here, indicate whether each material, system or method listed is relevant to your study. If you are not sure if a list item applies to your research, read the appropriate section before selecting a response.

Materials & experimental systems

n/a	Involved in the study
☒	☐ Antibodies
☒	☐ Eukaryotic cell lines
☒	☐ Palaeontology
☒	☐ Animals and other organisms
☒	☐ Human research participants
☒	☐ Clinical data

Methods

n/a	Involved in the study
☒	☐ ChIP-seq
☒	☐ Flow cytometry
☒	☐ MRI-based neuroimaging
