# Supplementary material for: Adaptive responses of animals to climate change are most likely insufficient
Source: Nat Commun. 2019 Jul 23;10:3109. doi: 10.1038/s41467-019-10924-4 (PMC6650445; doi:10.1038/s41467-019-10924-4)
Supplement: Supplementary file 4 — Description of Additional Supplementary Files [file 41467_2019_10924_MOESM4_ESM.pdf]

**Description of Additional Supplementary Files**

File Name: Supplementary Data 1
Description: Model estimates and their standard errors obtained from mixedeffects meta-analyses. The models were fitted separately for each climatic factor in both PRCS ('Phenotypic Responses to Climate with Selection data') and PRC ('Phenotypic Response to Climate') datasets. For details on the levels of the fixed effects see Methods.

File Name: Supplementary Data 2
Description: Significance of the effects tested with mixed-effects meta-analyses. Significance was estimated with an asymptotic likelihood ratio chi-square test (LRT) comparing the model that includes the effect specified in the column 'Effect' to the corresponding model without this effect. Significant effects ($p < 0.05$) are highlighted in bold and marginally significant ones ($p < 0.10$) in italic. For each model we also show the variance of the random effects for study and publication identity.

File Name: Supplementary Data 3
Description: References for publication identities used in the PRC ('Phenotypic Response to Climate') dataset and referred to in Figs. 2-4 and Supplementary Figs. 3, 7 and 8. For each publication, we indicate taxa, trait(s) and climatic factor(s) for which the data were reported and whether selection data were reported (if 'Yes', then the study also belongs to the PRCS 'Phenotypic Responses to Climate with Selection data' dataset).

File Name: Supplementary Data 4
Description: Metadata for each study in the PRC dataset and associated effect sizes for each of the three conditions.
